# Neurodevelopmental Trajectories at 3 Years: Insights From the NASCITA Italian Birth Cohort

**DOI:** 10.1155/ijpe/9936886

**Published:** 2026-03-09

**Authors:** Giulia Segre, Elisa Roberti, Rita Campi, Antonio Clavenna, Maurizio Bonati

**Affiliations:** ^1^ Department of Medical Epidemiology, Laboratory of Child Health and Development Epidemiology, Istituto di Ricerche Farmacologiche Mario Negri IRCCS, Milan, Italy, marionegri.it

**Keywords:** child development, cohort study, early intervention, neurodevelopmental disorders, risk factors, warning signs

## Abstract

**Aim:**

Early childhood development is critically influenced by exposure to stressful life events. Identifying children with warning signs (WS) for developmental disorders early is essential for timely intervention.

**Methods:**

Family pediatricians evaluated the neurodevelopment of 148 children from the NASCITA cohort using the CDC′s Learn the Signs, Act Early Milestones checklist, whereas parents completed the Strengths and Difficulties Questionnaire (SDQ). Univariate and multivariate analyses evaluated associations between WS and maternal characteristics, data concerning pregnancy, delivery and the newborns′ health, sleep disturbances and life habits.

**Results:**

A total of 14% of children showed WS for developmental disorders at 36 months, a lower percentage than at 24 months (15.8%): for two out of three children, WS disappeared between 24 and 36 months. Persistent WS were noted in 5% of children. Key risk factors identified include older maternal age at delivery (OR 8.93, 95% CI: 1.87–42.62) and maternal unemployment (OR 4.75, 95%CI: 1.40–16.09). Reading aloud emerged as a protective practice, emphasizing its potential in early interventions.

**Conclusions:**

These results highlight the need for continuous monitoring of WS and the importance of positive parental practices in mitigating developmental risks. Early identification by primary care practitioners is crucial in addressing developmental concerns early and improving long‐term outcomes.

## 1. Introduction

The first 3 years of life represent a critical period in which early experiences and potential risk factors shape long‐term outcomes [1]. First, maternal (e.g., smoke or alcohol consumption during pregnancy and maternal BMI), perinatal (e.g., preterm birth and complications at birth), and genetic factors need to be considered [2–5].

Second, lifestyle domains such as family characteristics, sociodemographic and health‐related factors (e.g., presence of chronic conditions in the family) and geographical area of residence are crucial [6, 7]. Lastly, the first 3 years of life can be characterized by a wide range of habits and practices, among which we can name sleep quality and bedtime routines, breastfeeding, reading aloud, outdoor activities, and exposure to screens or digital devices [8–12].

Developmental surveillance is fundamental, and it should assess all these aspects alongside clinical ones and parental concerns [13]. Monitoring growth, feeding, sleep habits, and motor, cognitive, socioemotional, and communicative abilities can reveal delays or difficulties in achieving milestones, which may indicate neurodevelopmental disorders [14]. For instance, early signs of language impairment can be early features of later language disorders and autism spectrum disorder [15]. Early preterm children may also exhibit slower gross motor skills and visual maturation, later followed by neurodevelopmental dysfunctions [16]. Lastly, mental health problems can occur in preschool children [17] with a prevalence up to 25% [18, 19], increasing the risk of mental disorders later in life [20–23]. Common risk factors for mental health problems include difficult temperament, being male, and having parents with poor physical health [24–26]. These delays and early difficulties are called warning signs (WS) [27, 28].

On the other hand, many are the protective factors that can improve developmental outcomes, such as supportive practices embedded in the nurturing care concept (e.g., no alcohol, no smoking, reading aloud, and listening to music in pregnancy and in the first months after birth, tummy time, exclusive breastfeeding for at least 6 months, reading aloud to children, time spent outdoors, minimizing screen exposure, and having bedtime routines) [29].

Given these premises, close monitoring of development in the first years of life is crucial to identify children with WS. Early intervention and support for the child and family are essential, as children′s development is particularly affected by exposure to environmental events [30].

In Italy, the childhood health plan calls for at least six screenings within the first 6 years of life (well‐child visits are suggested at 45 days, 3, 6, 9,12, 18, and 36 months as well as 6,10, and 14 years). These visits allow collecting data on growth and well‐being, with family pediatricians (FPs) playing a key role in this screening [31]. The NASCITA study, which began in 2020, collected data during these visits using integrated tools and questionnaires to evaluate an Italian cohort′s first 3 years of life [32]. It explored several aspects of children′s early development and parental practices, incorporating FPs′ and parents′ perspectives. During visits, FPs used routine tools (e.g., measuring weight, length, and head circumference) alongside additional tools to monitor psychomotor development, milestones achievement, social skills, WS, and parenting stress. Further details on screening methods are reported elsewhere [33]. Briefly, we considered covariates spanning maternal, perinatal, and lifestyle domains. Maternal and family characteristics included sociodemographic and health‐related factors such as parental age, education, employment and marital status, as well as parity, geographical area of residence, and the presence of chronic conditions. We also accounted for pregnancy‐ and birth‐related variables, including maternal pre‐pregnancy BMI, gestational weight gain, mode and timing of delivery, and neonatal health indicators such as prematurity or birth complications, alongside early practices like skin‐to‐skin contact. Beyond these perinatal aspects, we considered the occurrence of sleep disturbances during the first 3 years of life and a wide range of lifestyle and caregiving practices, such as breastfeeding, reading aloud, tummy time, bedtime routines, outdoor activities, and exposure to screens or digital devices.

At the 24‐month assessment, a focus on neurodevelopment and possible WS linked to risk for neurodevelopmental disorders was implemented. Of the 435 children and their parents who participated, 69 (15.8%) presented WS based on the parental and/or FPs′ assessments. Thirty‐three children (including seven without WS) were referred to a child psychiatrist, and 16 were diagnosed with a developmental disorder (primarily language delays) [34]. The study′s strength was its integration of parental and FPs perspectives, demonstrating the feasibility of a comprehensive child assessment. However, it left unanswered whether detected WS would resolve spontaneously or require further attention, highlighting the need for additional data collected at later stages.

This study is a follow‐up at 36 months within the same NASCITA cohort subsample of the neurodevelopment screening. It is aimed at (1) identifying children at risk for emotional or behavioral WS at 36 months, (2) comparing findings with the 24‐month assessment, and (3) exploring risk and protective factors associated with WS.

## 2. Methods

**Figure 1:**
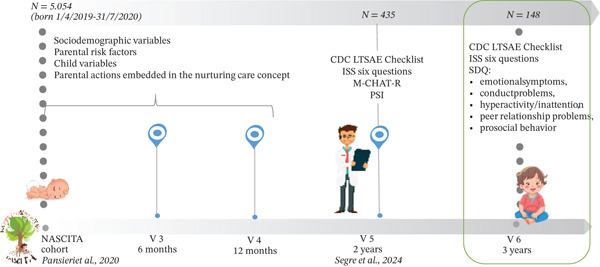
Study design and variables collected at the different time points up to the 3‐year visit.

As a follow‐up of the 24‐month assessment of the children in the NASCITA cohort (Figure 1) [34], each child was evaluated at the 36‐month screening as follows:

• *FPs assessment*. As was done at 24 months, FPs completed the routine questions on physical growth and health care checks, the CDC′s Learn the Signs, Act Early Milestones (LTSAE) 3‐year checklist. Although this milestone checklist cannot be considered a developmental screening tool, it effectively promotes developmental monitoring and encourages conversations between parents and healthcare providers about child development [35]. Following the Istituto Superiore di Sanità (Italian National Institute of Health—ISS) recommendations, two questions were added to the FP assessment for a total of 38 items. FPs identified toddlers as at risk of developmental disorders (“positive”) if the total number of failed items was ≥ 12. This cutoff was defined by classifying scores in percentiles and considering the ones above the 95th percentile as a WS, as commonly done with developmental tools [36, 37].
• *Parent assessment of the child′s development*. Parents filled in the Strengths and Difficulties Questionnaire (SDQ). In the previous visit (at 24 months), the Modified Checklist for Autism in Toddlers, Revised (M‐CHAT‐R) was employed. However, that specific tool is intended for toddlers between 16 and 30 months of age [38]. For this reason, the SDQ was chosen for the 36‐month screening, which shares with the M‐CHAT‐R that it is an instrumental tool designed to identify developmental problems in early childhood. Indeed, the SDQ is a highly structured, multi‐informant questionnaire for parents, teachers, and children designed to help identify children aged 3–16 years who may have behavioral or emotional problems. The questionnaire, which is quick and easy to answer, comprises 25 items grouped into five scales: emotional symptoms, conduct problems, hyperactivity/inattention, peer relationship problems, and prosocial behavior. The scores of the first four subscales of the SDQ are summed together to obtain a total difficulties score ranging from 0 to 40. A score categorized as 0–13 is classified as normal, whereas SDQ scores between 14 and 40 are considered borderline/abnormal (in this study, defined as “at risk for WS for emotional or behavioral problems”) [39, 40]. For this study, parents filled in the Italian version of the SDQ for the youngest preschool children, which has already been used and validated in Italy [41, 42] as well as in other countries [43–45].

The primary outcome measure was the prevalence of children with WS for developmental disorders. Univariate and logistic regression analyses were performed to evaluate which variables were associated with a higher likelihood of WS.

Moreover, three groups of children were identified: (1) those presenting WS both at 24 and 36 months of age (persistent WS); (2) those presenting WS only at 24 or 36 months of age; and (3) those never presenting WS.

The characteristics of the three groups were compared using chi‐square test and chi‐square for trends, when applicable. Variables previously associated with a child presenting WS were selected as covariates [33, 46] and added into a univariate model. They included maternal characteristics, data concerning pregnancy, delivery and the newborn, sleep disturbances, and lifestyle habits. A detailed list is provided in the File S1.

Categorical variables were summarized using proportions and frequency distributions, and associations were tested using chi‐square or chi‐square for trend and Fisher′s exact test where applicable. Logistic stepwise regression analyses were used to identify variables associated with being at risk for behavioral and emotional problems. The least absolute shrinkage selection operator (LASSO) regression analysis was employed to identify the most significant variables, and multivariate logistic regression analysis was subsequently conducted to validate the selected predictive factors and establish a predictive model based on the results of the LASSO regression analysis. R Version 4.5.1 was used to complete the LASSO regression model and construct the nomogram, as well as to plot the ROC curves, calibration curves, and decision curves. We used pairwise deletion for missing data so that all variables were used. The Hosmer–Lemeshow test was used to determine the goodness of fit of the logistic regression model. Data were analyzed using SAS software, Version 9.4 (SAS Institute, North Carolina, United States).

The study was conducted in accordance with the Declaration of Helsinki (1964), approved by the Fondazione IRCCS Istituto Neurologico Carlo Besta′s Ethics Committee (February 6, 2019, Protocol No. 59, June 9, 2021, Protocol No. 85), and written informed consent has been appropriately obtained from all parents.

An earlier Italian‐language dissemination of the findings was published for primary care practitioners in Italy [33]. The present manuscript substantially extends this material by providing a comprehensive, peer‐reviewed analysis.

## 3. Results

**Figure 2:**
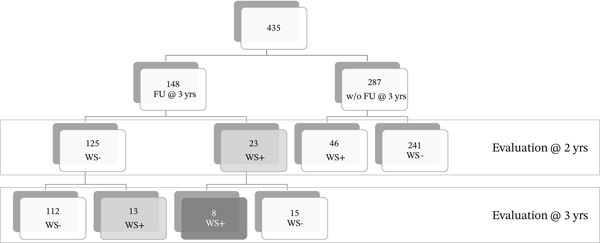
Flowchart reporting the number of children with (FU) or without a follow‐up (w/o FU) from the 2‐ to the 3‐year visit. It is also specified whether children had warning signs (WS+) or no warning signs (WS−).

Data concerning the assessment at 36 months of age were available for 148 infants (78 male and 70 female). These 148 children are representative of the original sample for sociodemographic characteristics, which have already been described elsewhere [34]. It was not possible to collect further information for the other 287 toddlers due to FP and/or parental difficulties in continuing the neurodevelopment evaluation (e.g., FPs′ retirement, families who moved or dropped out of the study) (Figure 2). However, an analysis was conducted to compare the characteristics of the 148 children who participated versus 287 who did not participate in the follow‐up assessment at 36 months. No significant differences emerged (all *p* > 0.1; results in Table S2).

### 3.1. WS at 36 Months

From the FP assessment and the parental SDQ questionnaire combined, 21 toddlers (14%) presented WS at the 36‐month assessment. In particular, four children had WS only in the FP assessment, 15 only in the SDQ, and two in both assessments; 127 were not at risk at the 3‐year assessment.

In the present sample, the median score of failed items at the SDQ was 7 (interquartile range 4.5–10). Considering the different subscales of SDQ, higher percentages (41.2%) of at‐risk children were found in the items included in the conduct problem scale, followed by the peer relationships problems scale (24.3%). Lower percentages were reported in the prosocial behavior (17.6%), hyperactivity/inattention (10.1%), and emotional symptoms (4.7%) subscales.

Older maternal age at delivery (*p* = 0.04), maternal unemployment (*p* = 0.04), and not reading aloud to children (*p* = 0.02) emerged as risk factors for possible WS in the univariate analysis (Table S3). The logistic regression (Table 1) confirmed that older (> 39 years) maternal age (OR: 7.85, 95% CI: 1.15–60.90) is associated with a greater likelihood of WS, in addition to maternal unemployment (OR: 4.64, 95% CI: 1.08–21.92) and not reading aloud to children (OR: 4.43, 95% CI: 1.29–16.83).

**Table 1:** Logistic regression model evaluating the variables associated with the presence of warning signs at 3 years of age.

	OR (95% IC)	*p*
Maternal age at delivery		
< 35 versus 30–34	0.26 (0.03–1.96)	0.2239
35–39 versus 30–34	1.54 (0.33–7.41)	0.5807
> 39 versus 30–34	7.85 (1.15–60.90)	**0.0384**
Maternal employment status: no versus yes	4.64 (1.08–21.92)	**0.0416**
Reading aloud to children: no versus yes	4.43 (1.29–16.83)	**0.0211**

*Note:* Accuracy: 88.4%; sensitivity: 66.7%. Statistically significant results at the 0.05 level are shown in bold.

### 3.2. Follow‐Up of Children With WS at 24 Months of Age

Of the 69 children with WS at the 24‐month assessment, 23 were followed up at the 3‐year assessment (four of whom received a formal diagnosis between 2 and 3 years of age: two language delays and two global developmental delays with specific language impairment). For eight of them, the WS persisted at 36 months (one was among the four with a formal diagnosis) (Figure 2).

A total of eight children had persistent WS (Group 1), 28 only at 24 or 36 months of age (Group 2), and 112 never presented WS (Group 3). Differences between these three groups emerged for the following variables: paternal chronic conditions, child gender, maternal employment status, and reading aloud (Table 2). In particular, being male (*p* = 0.04) increased the likelihood of being at risk for WS both in Groups 1 and 2; differences in paternal chronic conditions were mainly found between Group 2 versus 3 (*p* = 0.03), whereas having an unemployed mother was found to be a risk factor only for Group 1 (*p* = 0.02), with no differences when comparing Group 2 versus 3. Reading aloud to children emerged as a protective factor for child development at the 24‐ and 36‐month assessments (*p* = 0.005). The proportion of parents reading aloud significantly increased from Group 1 to Group 3 ($\chi_{t}^{2}=11.3$; *p* = 0.0008). Unfortunately, it was not possible to perform a multivariate analysis due to the limited sample size.

**Table 2:** Comparison of the characteristics of children with warning signs at both the 2‐ and 3‐year well‐child visit, versus children with warning signs at one visit only, and children without warning signs.

		Children at risk at 24 and 36 months (*N* = 8)	Children at risk at 24 or 36 months (*N* = 28)	Children never at risk (*N* = 112)	Total	*p* value
Geographical area of residence	North	6 (75.0)	19 (67.9)	82 (73.2)	107 (72.3)	0.97
	Center	1 (12.5)	4 (14.3)	13 (11.6)	18 (12.2)	
	South	1 (12.5)	5 (17.9)	17 (15.2)	23 (15.5)	

Both parents are Italian	Yes	5 (62.5)	24 (85.7)	101 (90.2)	130 (87.8)	0.074
	No	3 (37.5)	4 (14.3)	11 (9.8)	18 (12.2)	

Maternal age at delivery	< 35	3 (37.5)	13 (46.4)	67 (59.8)	83 (56.1)	0.26
	> = 35	5 (62.5)	15 (53.6)	45 (40.2)	65 (43.9)	

Paternal age at delivery	< 35	1 (12.5)	9 (32.1)	48 (43.2)	58 (39.5)	0.17
	> = 35	7 (87.5)	19 (67.9)	63 (56.8)	89 (60.5)	
	Missing data	—	—	1	1	

Maternal educational level^a^	High	7 (87.5)	26 (92.9)	99 (88.4)	132 (89.2)	0.79
	Low	1 (12.5)	2 (7.1)	13 (11.6)	16 (10.8)	

Paternal educational level^a^	High	5 (62.5)	24 (85.7)	92 (82.9)	121 (82.3)	0.31
	Low	3 (37.5)	4 (14.3)	19 (17.1)	26 (17.7)	
	Missing data	—	—	1	1	

Maternal employment status	Employed	3 (37.5)	23 (82.1)	89 (79.5)	115 (77.7)	**0.019**
	Unemployed	5 (62.5)	5 (17.9)	23 (20.5)	33 (22.3)	

Marital status	With partner	—	—	1 (0.9)	1 (0.7)	1.00
	Single mother	8 (100.0)	28 (100.0)	111 (99.1)	147 (99.3)	

Maternal chronic conditions	Yes	2 (25.0)	5 (17.9)	27 (24.1)	34 (23.0)	0.77
	No	6 (75.0)	23 (82.1)	85 (75.9)	114 (77.0)	

Paternal chronic conditions	Yes	2 (25.0)	10 (35.7)	16 (14.4)	28 (19.0)	**0.034**
	No	6 (75.0)	18 (64.3)	95 (85.6)	119 (81.0)	
	Missing data	—	—	1	1	

Pre‐pregnancy BMI	Underweight	—	2 (7.1)	10 (8.9)	12 (8.1)	0.94
	Normal	6 (75.0)	17 (60.7)	74 (66.1)	97 (65.5)	
	Overweight or obese	2 (25.0)	9 (32.1)	28 (25.0)	39 (26.4)	

Gestational weight gain	Below	4 (50.0)	10 (35.7)	33 (29.7)	47 (32.0)	0.42
	Normal	4 (50.0)	10 (35.7)	49 (44.1)	63 (42.9)	
	Over	—	8 (28.6)	29 (26.1)	37 (25.2)	
	Missing data	—	—	1	1	

Delivery during first pandemic wave^b^	Yes	7 (87.5)	24 (85.7)	98 (87.5)	129 (87.2)	0.89
	No	1 (12.5)	4 (14.3)	14 (12.5)	19 (12.8)	

Primiparous	Yes	3 (37.5)	19 (67.9)	50 (45.0)	72 (49.0)	0.08
	No	5 (62.5)	9 (32.1)	61 (55.0)	75 (51.0)	
	Missing data	—	—	1	1	

C‐section delivery	Yes	4 (50.0)	10 (35.7)	29 (25.9)	43 (29.1)	0.24
	No	4 (50.0)	18 (64.3)	83 (74.1)	105 (70.9)	

Healthy newborn	Yes	7 (87.5)	23 (82.1)	101 (90.2)	131 (88.5)	0.42
	No	1 (12.5)	5 (17.9)	11 (9.8)	17 (11.5)	

Newborn gender	Female	1 (12.5)	10 (35.7)	59 (52.7)	70 (47.3)	**0.04**(0.007)^c^
	Male	7 (87.5)	18 (64.3)	53 (47.3)	78 (52.7)	

Skin to skin contact at birth	Yes	5 (62.5)	18 (64.3)	90 (80.4)	113 (76.4)	0.13
	No	3 (37.5)	10 (35.7)	22 (19.6)	35 (23.6)	

Child sleeping disorders (from 6 months to 2 years)	Yes	5 (62.5)	12 (42.9)	57 (51.4)	74 (50.3)	0.56
	No	3 (37.5)	16 (57.1)	54 (48.6)	73 (49.7)	
	Missing data	—	—	1	1	

Mother smoker in pregnancy	Yes	7 (87.5)	27 (96.4)	101 (91.0)	135 (91.8)	0.42
	No	1 (12.5)	1 (3.6)	10 (9.0)	12 (8.2)	
	Missing data	—	—	1	1	

Mother consuming alcohol in pregnancy	Yes	7 (87.5)	25 (89.3)	92 (82.9)	124 (84.4)	0.83
	No	1 (12.5)	3 (10.7)	19 (17.1)	23 (15.6)	
	Missing data	—	—	1	1	

Exclusive breastfeeding for at least 6 months	Yes	1 (25.0)	3 (13.0)	23 (27.7)	27 (24.5)	0.30
	No	3 (75.0)	20 (87.0)	60 (72.3)	83 (75.5)	
	Missing data	4	5	29	38	

Reading aloud to children	Yes	2 (25.0)	12 (42.9)	76 (67.9)	90 (60.8)	**0.005** (0.0008)^c^
	No	6 (75.0)	16 (57.1)	36 (32.1)	58 (39.2)	

Tummy time	Yes	4 (50.0)	24 (85.7)	92 (82.1)	120 (81.1)	0.06
	No	4 (50.0)	4 (14.3)	20 (17.9)	28 (18.9)	

Bedtime routine	Yes	1 (12.5)	4 (15.4)	27 (27.6)	32 (24.2)	0.32
	No	7 (87.5)	22 (84.6)	71 (72.4)	100 (75.8)	
	Missing data	—	2	14	16	

Outdoor activities	Yes	7 (87.5)	25 (96.2)	105 (94.6)	137 (94.5)	0.59
	No	1 (12.5)	1 (3.8)	6 (5.4)	8 (5.5)	
	Missing data	—	2	1	3	

Frequency of screen exposure	Low	2 (25.0)	4 (15.4)	14 (12.6)	20 (13.8)	0.44
	Medium/high	6 (75.0)	22 (84.6)	97 (87.4)	125 (86.2)	
	Missing data	—	2	1	3	

Frequency of interaction with devices	Low	4 (50.0)	9 (34.6)	32 (29.1)	45 (31.3)	0.43
	Medium/high	4 (50.0)	17 (65.4)	78 (70.9)	99 (68.8)	
	Missing data	—	2	2	4	

TV on time in the home^d^	Yes	4 (80.0)	17 (89.5)	68 (89.5)	89 (89.0)	0.57
	No	1 (20.0)	2 (10.5)	8 (10.5)	11 (11.0)	
	Missing data	3	9	36	48	

*Note:* Statistically significant results at the 0.05 level are shown in bold.

^a^Educational level: low—no schooling or primary versus high—secondary school or university.

^b^Delivery during first pandemic wave: Yes—delivery between 24/02/2020 and 31/07/2020; No—delivery between 01/04/2019 and 23/02/2020.

^c^ *p* value of chi‐square for trend test.

^d^TV on time refers to the adoption of a positive parental approach (i.e., less than 4 hours/day at 12, 24 and 36 months).

## 4. Discussion

At the 36‐month follow‐up, a 14% prevalence of WS was observed, only slightly lower than the 15.8% reported at 24 months. Six out of ten children were incident cases (signs not present at the previous evaluation), whereas nearly two‐thirds of children with WS at 24 months no longer exhibited them at 36 months, consistent with findings that children may reach developmental milestones with delays [47, 48]. The incident cases confirm that new developmental concerns may emerge after the first 2 years of life. This is not surprising if we consider the changes that occur around 3 years of age: children transition toward more and more complex language, social interaction, self‐regulation, and fine motor abilities [49]. It is therefore natural that, alongside these new milestones, some developmental delays or parental concerns may become apparent. However, 5% of children had persistent WS, suggesting that they were at higher risk for developmental disorders. This aligns with literature‐reported prevalence rates for neurodevelopmental disorders, such as autism spectrum disorders (0.70%–3%), intellectual disorders (0.63%–1.4%), and learning disabilities (3%–6.4%) [50–52].

Young maternal age at delivery, maternal employment, and reading aloud to children were associated with a reduced likelihood of developmental disorders at 36 months. The impact of maternal age on developmental vulnerability has been widely studied, with very young (18–24) and advanced (35–39) maternal ages correlated with ADHD and learning disability risks [53, 54]. Given the nature of these links found in the literature and the later onset of socioemotional and cognitive concerns, which often become more apparent after 3 years, it is not entirely surprising that maternal age emerged as a risk factor at 36 but not at 24 months. Similarly, maternal unemployment has been associated with milestone achievement failure [55]. Reading aloud to children consistently emerged as a key protective factor. This result aligns with previous evidence identifying reading aloud as an effective strategy to improve language development and other emerging literacy skills [56, 57]. One study reported that reading aloud and playing reduced behavior problems such as hyperactivity and enhanced socioemotional development in infants and toddlers [9]. Programs promoting reading aloud also improved parent–child interactions and child language and cognitive development across diverse cultural and educational backgrounds [58, 59]. Our findings underscore its effectiveness in reducing WS likelihood over time, encouraging its inclusion in other intervention programs.

At the previous 24‐month assessment, being male and having sleep disorders were associated with WS, whereas reading aloud was a protective factor [33]. Although reading aloud was consistently protective at both 24 and 36 months, male gender and sleep disorders influenced WS only at 24 months.

Considering the eight children with persistent WS, boys still had more WS, as reported in other studies [60, 61]. Moreover, having a paternal chronic condition increases the likelihood of persistent WS, aligning with evidence linking paternal health to children′s developmental difficulties [62]. Children with these characteristics warrant closer developmental monitoring. One noteworthy observation on these eight children with persistent WS is that only one out of the four children who received a diagnosis in the previous year still falls in this WS group 1 year later, suggesting that once a diagnosis is established and support is provided, many children may move out of the WS category by age 3.

Lastly, the SDQ revealed higher percentages of at‐risk children on the conduct and peer relationship problem scales. Although no research is available on the importance of these specific subscales, they are related to interactive difficulties compared with other scales. The failure to reach age‐appropriate social milestones might lead to an increased risk for persistent problems, such as aggressive and oppositional behavior and emotional difficulties [63]. Pediatricians should prioritize these areas in developmental monitoring [64].

The small sample size and low number of children with WS limited a robust comparison between children with persistent and occasional WS. However, some relevant differences, such as maternal employment status and reading aloud, still emerged. Unfortunately, data from 287 toddlers were unavailable at 36 months, but their characteristics and WS prevalence (16%) were similar to the 148 children in the follow‐up (15.5%), suggesting the sample′s representativeness (Figure 2). One limitation to keep in mind is that although the characteristics of the children followed up and not followed up were similar, an attrition bias may still be present because of other characteristics.

In conclusion, this study emphasizes the importance of early identification and intervention for WS, with shared vigilance between parents and pediatricians. Parental attitudes and practices can be a protective factor to promote optimal early child development [65], and pediatricians play a crucial role in supporting parents, reinforcing their confidence, creating knowledge, and providing another observation point. Lastly, policies should address maternal employment challenges and promote parental practices such as reading aloud, which may reduce developmental disorder risks and persistent WS.

**Nomenclature**
ADHD: attention deficit hyperactivity disorder
BMI: body mass index
FP: family pediatrician
ISS: Istituto Superiore di Sanità (Italian National Institute of Health)
LTSAE: Learn the Signs, Act Early Milestones
M‐CHAT‐R: Modified Checklist for Autism in Toddlers, Revised
NICU: neonatal intensive care unit
SDQ: Strengths and Difficulties Questionnaire
WS: warning signs

## Funding

This work was supported by resources from the Laboratory of Epidemiology of Child Health and Development Epidemiology and by an economic contribution by the Associazione Amici del Mario Negri (Grant number: N/A).

## Disclosure

The Associazione Amici del Mario Negri had no role in the design and conduct of the study. None of the authors is a member of the Associazione Amici del Mario Negri.

## Conflicts of Interest

The authors declare no conflicts of interest.

## Supporting information


**Supporting Information** Additional supporting information can be found online in the Supporting Information section. File S1: list and detailed explanation of the covariates included in the study. Table S2: Comparison of the characteristics of 148 children who participated in the follow‐up assessment at 36 months versus 287 who did not participate. Table S3: Comparison of characteristics of children with versus without warning signs at the 36‐month well‐child visit.

## Data Availability

Data are available on request.
