# Supplementary material for: Neurodevelopmental Trajectories at 3 Years: Insights From the NASCITA Italian Birth Cohort
Source: Int J Pediatr. 2026 Mar 9;2026:9936886. doi: 10.1155/ijpe/9936886 (PMC12969220; doi:10.1155/ijpe/9936886)
Supplement: Supplementary file 1 — Supporting Information Additional supporting information can be found online in the Supporting Information section. File S1: list and detailed explanation of the covariates included in the study. Table S2: Comparison of the characteristics of 148 children who participated in the follow‐up assessment at 36 months versus 287 who did not participate. Table S3: Comparison of characteristics of children with versus without warning signs at the 36‐month well‐child visit. [file IJPE-2026-9936886-s001.docx]

**Neurodevelopmental trajectories at 3 years: Insights from the NASCITA Italian birth cohort.**

**SUPPORTING INFORMATION**

[Supporting Information S1: list and detailed explanation of the covariates included in the study. 2](#_Toc209712569)

[Supporting Information S2: Comparison of the characteristics of 148 children who participated in the follow-up assessment at 36 months versus 287 who did not participate 3](#_Toc209712570)

[Supporting Information S3: Comparison of characteristics of children with versus without warning signs at the 36-months well-child visit. 5](#_Toc209712571)

# Supporting Information S1: list and detailed explanation of the covariates included in the study.

*- Maternal characteristics:* geographical area of residence (North/Centre/South), both parents Italian (yes/no), age of the parents at delivery (<35 vs ≥35 years), parental educational level (low: no schooling or primary school; high: secondary school or university), maternal employment status, marital status, presence of parental chronic conditions (yes/no), and parity (primiparous yes/no).

*- Data concerning pregnancy, delivery and newborn:* pre-pregnancy BMI (Mothers were grouped according to their pre-pregnancy BMI into three categories, underweight (≤18.5), normal (18.6-24.9), and overweight or obese (≥25.0) , gestational weight gain (below/normal/over compared with to the weight variations recommended by the Institute of Medicine criteria), delivery during the first wave of COVID-19 pandemic (*Yes: delivery between 24/02/2020 and 31/07/2020; No: delivery between 01/04/2019 and 23/02/2020)*, C-section delivery, gender of the neonate, healthy newborn (yes/no; Yes: toddlers who were not born preterm and/or low birth weight, had no malformations at birth, and were not admitted to the NICU in the postnatal period), skin to skin contact at birth.

*- Occurrence of sleep disturbances:* If parents reported a sleep disturbance (mainly recurrent night awakenings) in at least one of the visits (12, 24, 36 months), the variable was “Yes”, otherwise it was “No”.

*- Lifestyle habits:*

- Mother smoker in pregnancy: yes/no.
- Mother consuming alcohol in pregnancy: yes/no.
- Exclusive breastfeeding for at least 6 months.
- Reading aloud to children: “Yes” if a book had been read to the child in the two weeks prior to *each* of the three visits (6, 12 and 24 months).
- Tummy time: considered adequate if the child was placed belly down for any amount of time each day at 6 months (Pandolfini et al., 2024; Segre et al., 2024).
- Bedtime routine: ”Yes” if parents reported to read a book or a to sing a song both at 12 AND 24 months visit, otherwise it was “No” (Pandolfini et al., 2024; Segre et al., 2024).
- Outdoor activities: mean number of daily hours spent outdoor by parents and infant (<1: score=1; 1-3: score=2; >3: score=3) were collected at 12, 24 and 36 months visits. The scored obtained in each visit was summed to obtain an overall score. The overall frequency was categorized in: low (total score ≤4) or Medium/high (total score > 4).
- TV-on time in the home: ”Yes” if < 4 hours per day at 12, 24 and 36 months of age visits, otherwise “No”.
- Frequency of screen exposure in watching video for each visit (12 24, 36 months) was evaluated as: Never=1, Sometimes=2, Daily =3. The scored obtained in each visit was summed to obtain an overall score. The overall frequency was categorized in: Low (total score ≤4) or Medium/high (total score >4).
- Frequency of Interaction with devices: At both the 12, 24, 36 months visits, the parent was asked to indicate whether the child interacts with smartphone/tablet: (1 = never, 2 = sometimes, 3 = often). The scored obtained in each visit was summed to obtain an overall score. The overall frequency was categorized in: Low (total score ≤4) or Medium/high (total score > 4).

# Supporting Information S2: Comparison of the characteristics of 148 children who participated in the follow-up assessment at 36 months versus 287 who did not participate

|  |  | **Participating** | **Not participating** | **p-value** |
| --- | --- | --- | --- | --- |
|  |  | N=148 | N=287 |  |
| Geographical area of residence | North | 107 (72.3) | 217 (75.6) | 0.31 |
|  | Center | 18 (12.2) | 22 (7.7) |  |
|  | South | 23 (15.5) | 48 (16.7) |  |
| Newborn gender | Female | 70 (47.3) | 133 (46.3) | 0.85 |
|  | Male | 78 (52.7) | 154 (53.7) |  |
| Maternal age group at delivery | <30 | 30 (20.3) | 66 (23.7) | 0.71 |
|  | 30-34 | 53 (35.8) | 100 (35.8) |  |
|  | 35-39 | 49 (33.1) | 79 (28.3) |  |
|  | >39 | 16 (10.8) | 34 (12.2) |  |
|  | Missing | - | 8 |  |
| Paternal age group at delivery | <30 | 18 (12.2) | 33 (11.9 | 0.24 |
|  | 30-34 | 40 (27.2) | 74 (26.7) |  |
|  | 35-39 | 41 (27.9) | 101 (36.5) |  |
|  | >39 | 48 (32.7) | 69 (24.9) |  |
|  | Missing | 1 | 10 |  |
| Maternal marital status | Single mother | 1 (0.7) | 9 (3.5) | 0.19 |
|  | With partner | 147 (99.3) | 277 (96.5) |  |
| Maternal educational level | High | 132 (89.2) | 247 (86.3) | 0.40 |
|  | Low | 16 (10.8) | 39 (13.7) |  |
|  | Missing | - | 1 |  |
| Paternal educational level | High | 121 (82.3) | 222 (78.7) | 0.38 |
|  | Low | 26 (17.7) | 60 (21.3) |  |
|  | Missing | 1 | 5 |  |
| Both parents Italian | Yes | 130 (87.8) | 256 (89.2) | 0.67 |
|  | No | 18 (12.2) | 31 (10.8) |  |
| Maternal employment status | Employed | 115 (77.7) | 213 (74.2) | 0.46 |
|  | Unemployed | 33 (22.3) | 73 (25.8) |  |
|  | Missing | - | 1 |  |
| Primiparous | Yes | 72 (49.0) | 149 (51.9) | 0.56 |
|  | No | 75 (51.0) | 138 (48.1) |  |
|  | Missing | 1 | 0 |  |
| C-section delivery | Yes | 43 (29.1) | 77 (26.8) | 0.62 |
|  | No | 105 (70.9) | 210 (73.2) |  |

# Supporting Information S3: Comparison of characteristics of children with versus without warning signs at the 36-months well-child visit.

|  |  | **Children at risk**  **(N=21)** | **Not at risk**  **(N=127)** | ***p*-value** |
| --- | --- | --- | --- | --- |
| Geographical area of residence | *North* | 14 (66.7) | 93 (73.2) | 0.52 |
|  | *Center* | 2 (9.5) | 16 (12.6) |  |
|  | *South* | 5 (23.8) | 18 (14.2) |  |
| Both parents Italian | *Yes* | 17 (81.0) | 113 (89.0) | 0.29 |
|  | *No* | 4 (19.0) | 14 (11.0) |  |
| Maternal age at delivery | *<30* | 2 (9.5) | 28 (22.0) | **0.01^a^** |
|  | *30-34* | 5 (23.8) | 48 (37.8) |  |
|  | *35-39* | 7 (33.3) | 42 (33.1) |  |
|  | *>39* | 7 (33.3) | 9 (7.1) |  |
| Paternal age at delivery | *< 30* | - | 18 (14.3) | 0.10^a^ |
|  | *30-34* | 5 (23.8) | 35 (27.8) |  |
|  | *35-39* | 5 (23.8) | 36 (28.6) |  |
|  | *>39* | 11 (52.4) | 37 (29.4) |  |
|  | *Missing data* | - | 1 |  |
| Maternal educational level^b^ | *High* | 18 (85.7) | 114 (89.8) | 0.70 |
|  | *Low* | 3 (14.3) | 13 (10.2) |  |
| Paternal educational level^b^ | *High* | 16 (76.2) | 105 (83.3) | 0.53 |
|  | *Low* | 5 (23.8) | 21 (16.7) |  |
|  | *Missing data* | - | 1 |  |
| Maternal employment status | *Employed* | 12 (57.1) | 103 (81.1) | **0.0225** |
|  | *Unemployed* | 9 (42.9) | 24 (18.9) |  |
| Marital status | *With partner* | - | 1 (0.8) | 1.00 |
|  | *Single mother* | 21 (100.0) | 126 (99.2) |  |
| Maternal chronic conditions | *Yes* | 4 (19.0) | 30 (23.6) | 0.78 |
|  | *No* | 17 (81.0) | 97 (76.4) |  |
| Paternal chronic conditions | *Yes* | 7 (33.3) | 21 (16.7) | 0.13 |
|  | *No* | 14 (66.7) | 105 (83.3) |  |
|  | *Missing data* | - | 1 |  |
| Pre-pregnancy BMI | *Underweight* | 1 (4.8) | 11 (8.7) | 0.77 |
|  | *Normal* | 15 (71.4) | 82 (64.6) |  |
|  | *Overweight or obese* | 5 (23.8) | 34 (26.8) |  |
| Gestational weight gain | *Below* | 9 (42.9) | 38 (30.2) | 0.18 |
|  | *Normal* | 10 (47.6) | 53 (42.1) |  |
|  | *Over* | 2 (9.5) | 35 (27.8) |  |
|  | *Missing data* | - | 1 |  |
| Delivery during first pandemic wave^c^ | *Yes* | 19 (90.5) | 110 (86.6) | 1.00 |
|  | *No* | 2 (9.5) | 17 (13.4) |  |
| Primiparous | *Yes* | 12 (57.1) | 60 (47.6) | 0.42 |
|  | *No* | 9 (42.9) | 66 (52.4) |  |
|  | *Missing data* | - | 1 |  |
| C-section delivery | *Yes* | 7 (33.3) | 36 (28.3) | 0.64 |
|  | *No* | 14 (66.7) | 91 (71.7) |  |
| Healthy newborn | *Yes* | 18 (85.7) | 113 (89.0) | 0.71 |
|  | *No* | 3 (14.3) | 14 (11.0) |  |
| Newborn gender | *Female* | 6 (28.6) | 64 (50.4) | 0.06 |
|  | *Male* | 15 (71.4) | 63 (49.6) |  |
| Skin to skin contact at birth | *Yes* | 14 (66.7) | 99 (78.0) | 0.27 |
|  | *No* | 7 (33.3) | 28 (22.0) |  |
| Child sleeping disorders (from 6 months to 2 years) | *Yes* | 12 (57.1) | 62 (49.2) | 0.50 |
|  | *No* | 9 (42.9) | 64 (50.8) |  |
|  | *Missing data* | - | 1 |  |
| Mother smoker in pregnancy | *Yes* | 19 (90.5) | 116 (92.1) | 0.68 |
|  | *No* | 2 (9.5) | 10 (7.9) |  |
|  | *Missing data* | - | 1 |  |
| Mother consuming alcohol in pregnancy | *Yes* | 18 (85.7) | 106 (84.1) | 1.00 |
|  | *No* | 3 (14.3) | 20 (15.9) |  |
|  | *Missing data* | - | 1 |  |
| Exclusive breastfeeding for at least 6 months | *Yes* | 3 (20.0) | 24 (25.3) | 0.94 |
|  | *No* | 12 (80.0) | 71 (74.7) |  |
|  | *Missing data* | 6 | 32 |  |
| Reading aloud to children | *Yes* | 8 (38.1) | 82 (64.6) | **0.02** |
|  | *No* | 13 (61.9) | 45 (35.4) |  |
| Tummy time | *Yes* | 16 (76.2) | 104 (81.9) | 0.55 |
|  | *No* | 5 (23.8) | 23 (18.1) |  |
| Bedtime routine | *Yes* | 3 (14.3) | 29 (26.1) | 0.25 |
|  | *No* | 18 (85.7) | 82 (73.9) |  |
|  | *Missing data* | - | 16 |  |
| Outdoor activities | *Yes* | 20 (95.2) | 117 (94.4) | 1.00 |
|  | *No* | 1 (4.8) | 7 (5.6) |  |
|  | *Missing data* | - | 3 |  |
| Frequency of screen exposure | *Low* | 4 (19.0) | 16 (12.9) | 0.49 |
|  | *Medium/high* | 17 (81.0) | 108 (87.1) |  |
|  | *Missing data* | - | 3 |  |
| Frequency of interaction with devices | *Low* | 8 (38.1) | 37 (30.1) | 0.46 |
|  | *Medium/high* | 13 (61.9) | 86 (69.9) |  |
|  | *Missing data* | - | 4 |  |
| TV-on time in the home^d^ | *Yes* | 9 (81.8) | 80 (89.9) | 0.35 |
|  | *No* | 2 (18.2) | 9 (10.1) |  |
|  | *Missing data* | 10 | 38 |  |

*^a^p-value of chi-square for trend test. ^b^Educational level: low: no schooling or primary versus high: secondary school or university.* ^c^*Delivery during first pandemic wave: Yes: delivery between 24/02/2020 and 31/07/2020; No: delivery between 01/04/2019 and 23/02/2020.*
